# To the Members of the Medical Society of the State of New York

**Published:** 1914-04

**Authors:** 


### To the Members of the Medical Society of the State of New York

The BUFFALO MEDICAL JOURNAL heartily endorses the invitation of the Medical Society of the County of Erie, that the State Society should meet in Buffalo in 1915. The precedent of holding at least occasional meetings at other cities than the Capital has already been established. It stimulates local interest, adds both to the attendance and the membership of the State Society and it is of interest to members from a distance. There is no danger that this plan will waive in any degree the claim of the Society to being an official part of the legal organization of the State. It is neither becoming nor necessary for a host or his representative to praise his home and its furnishings. Suffice it to say that Buffalo is readily accessible—and, for a larger number of the profession than any other city except the Metropolis, quickly accessible—that it can assure adequate accommodations, both as to hotels and meeting places, and that it possesses many points of interest, professional and otherwise.

We also endorse, editorially, personally and as a member of the State Society, the candidacy of Dr. Grover W. Wende for President. Owing to long personal friendship and professional acquaintance, and to the association of Dr. Wende with this JOURNAL, it would be still less becoming, as it is still less necessary to justify this endorsement. Dr. Wende, both professionally and on account of his devotion to the cause of medical organization, is a logical candidate, irrespective of the place of meeting of the Society. The acceptance of the invitation to meet in Buffalo renders it both natural courtesy and a practical convenience to select for the Presidency a local man, and Dr. Wende is the unanimous choice of the County Society.

And we just can't help adding that he is one of the best fellows who ever grew up in western New York.

